# Evaluation of ocular surface impairment in meibomian gland dysfunction of varying severity using a comprehensive grading scale

**DOI:** 10.1097/MD.0000000000016547

**Published:** 2019-08-02

**Authors:** Jiayu Fu, Yilin Chou, Ran Hao, Xiaodan Jiang, Yushi Liu, Xuemin Li

**Affiliations:** Department of Ophthalmology, Peking University Third Hospital, Beijing, P.R. China.

**Keywords:** corneal nerve, dendritic cell, in vivo confocal microscopy, meibomian gland dysfunction

## Abstract

This study aimed to propose a comprehensive grading scale to evaluate different clinical manifestations in patients with varying severity of meibomian gland dysfunction (MGD) and analyze the correlations between the parameters of ocular surface impairment in MGD.

A total of 63 patients with MGD were enrolled. Ten specific symptoms were evaluated each with a subjective score and total score was applied to grade the severity of MGD. Thirty-seven patients were diagnosed with mild, 19 with moderate, and 7 with severe MGD. Slit-lamp and keratography were used to assess the signs of ocular surface and meibomian gland (MG). In vivo confocal microscopy (IVCM) was performed to evaluate the corneal nerves and dendritic cells. The differences and correlations between symptoms, signs, and IVCM parameters were analyzed.

Dryness, foreign body sensation, asthenopia, and photophobia were the most common and severe symptoms in our patients. The severe MGD group showed worse MG expressibility, Meibum score, Meiboscore, MG score, and higher nerve reflectivity (*P* < .05). The mild MGD group showed higher nerve density (*P* < .05). Total symptom score was negatively correlated with nerve density (*r* = –0.374, *P* < .05), while positively correlated with nerve reflectivity and dendritic cell density (*r* = 0.332 and 0.288, respectively, *P* < .05). MG score was correlated with nerve reflectivity (*r* = 0.265, *P* < .05).

The comprehensive grading scale was suitable for evaluating clinical manifestations in MGD of varying severity. The relationship between the specific symptoms, signs, and IVCM results concerning whole ocular surface impairment could help elucidate MGD pathophysiology and benefit evaluation or treatment in the future.

## 1 Introduction

Considered to be a major cause of dry eye syndrome, meibomian gland dysfunction (MGD) is one of the most common ocular disorders encountered in ophthalmic clinics with prevalence ranging from 3.5% to 70.0% worldwide,^[1,2]^ and has been found to be 69.3% in China.^[3]^ The International Workshop on MGD defined the disease to be chronic, diffuse abnormality of the meibomian glands, leading to morphological, and/or functional alterations.^[4]^ Thus, MGD is commonly characterized by symptoms of eye irritation and typical changes of signs in the meibomian gland or the meibum, which is secreted to form a thin lipid layer to protect the tear film covering the ocular surface from evaporation.^[5]^ Moreover, recent studies have emphasized that the pathophysiologic alterations caused by MGD will eventually impair the tear film and eventually the whole ocular surface as the disease progresses.^[6–8]^ This calls for the ability to evaluate the severity of MGD with a more integrated system including the whole ocular surface morpho-functional unit to adjust treatment accordingly.

With its high prevalence rate and the significant damage caused by MGD, researchers have thus far attached great importance to the disease and made marked progress in developing grading scales to evaluate its severity. These scales include symptom questionnaires such as the Ocular Surface Disease Index or Standard Patient Evaluation of Eye Dryness^[9]^; sign examinations for the meibomian gland (i.e., lid margin abnormality, meibomian gland expression, and meibum quality), tear condition (i.e., tear break-up time, Schirmer test), or ocular surface (i.e., corneal fluorescein staining)^[10]^; and recently applied tests requiring elaborate equipment such as confocal microscopy^[11]^ and keratography^[12]^ offering information on microstructure. However, the clinical practice or scientific study of MGD remains challenging for the following reasons. First, most studies have used the traditional questionnaires designed for dry eye syndrome which limits the ability to differentiate MGD. And the evaluation was not refined to specific symptoms which make it difficult to realize precise medicine.^[13]^ Second, there are no accepted reliable systems to examine the signs of MGD despite various schemes having been proposed, and the results of the signs are not matched with the symptoms leading to much controversy.^[14]^ Moreover, recent studies did not assess the whole ocular surface morpho-functional unit in patients with MGD would definitely limit the further understanding of the disease.

We developed an integrated grading scale to evaluate the whole ocular surface morpho-functional unit impairment in patients with varying severity of MGD and further explored the relationships of the clinical manifestations through comparison and correlation. We investigated whether a comprehensive evaluation including the specific symptom questionnaire, the examinations for signs of the meibomian gland, tear quality, and ocular surface; and in vivo confocal microscopy (IVCM) analysis of the corneal nerves and inflammatory cells is suitable for assessing the severity of MGD and to adjust treatment accordingly.

## 2 Materials and methods

### 2.1 Patients

This cross-sectional study included 63 eyes in 63 patients with MGD recruited from the outpatient department of the Department of Ophthalmology at Peking University Third Hospital between April 2018 and August 2018. MGD was diagnosed according to China's DED diagnostic criteria^[15]^: any symptoms of ocular discomfort, at least one clinical sign of meibomian gland: abnormality of the lid margin feature (i.e., lid margin irregularity, vascular engorgement, glandular orifices obstruction), alteration in meibum quality and/or expressibility. If just a single eye meets the diagnosis standards, it will be selected; if both eyes meet diagnosis standards, the right eye will be selected. Exclusion criteria were: subjects unable to complete the questionnaire or understand the procedures; the presence of ocular or systemic disease involving the ocular surface; history of previous eye surgery; use of topical medications or contact lenses that may affect the ocular surface.

Patients completed our questionnaire for the assessment of specific symptoms and were further divided according to the total score of the symptoms, representing the severity of the disease into 3 groups: the mild MGD group (n = 37, Total score ≤30), the moderate MGD group (n = 19, 30 < Total score ≤ 60), and the severe MGD group (n = 7, Total score > 60). All patients were first diagnosed before receiving treatment when enrolled.

The study protocol was approved by the Human Research and Ethics Committee of Peking University Third Hospital, and the research was in compliance with the tenets of the Declaration of Helsinki. Written informed consent was obtained from each participant before enrollment.

### 2.2 Clinical evaluation

Each patient completed the 4 parts of the clinical examinations as shown in Table 1 in the following order: MGD Symptom Evaluation: our questionnaire with visual analogue scales of ten specific symptoms; tear and ocular surface evaluation: tear meniscus height (TMH), tear break-up time (TBUT), and corneal fluorescein staining (CFS); meibomian gland evaluation: lid marginal abnormality, meibomian gland expressibility, the property of meibom and MG dropout; and IVCM analysis of the central corneal sub-basal nerves and inflammatory cells. All procedures were performed by the same ophthalmologist in a dark room.

**Table 1:**
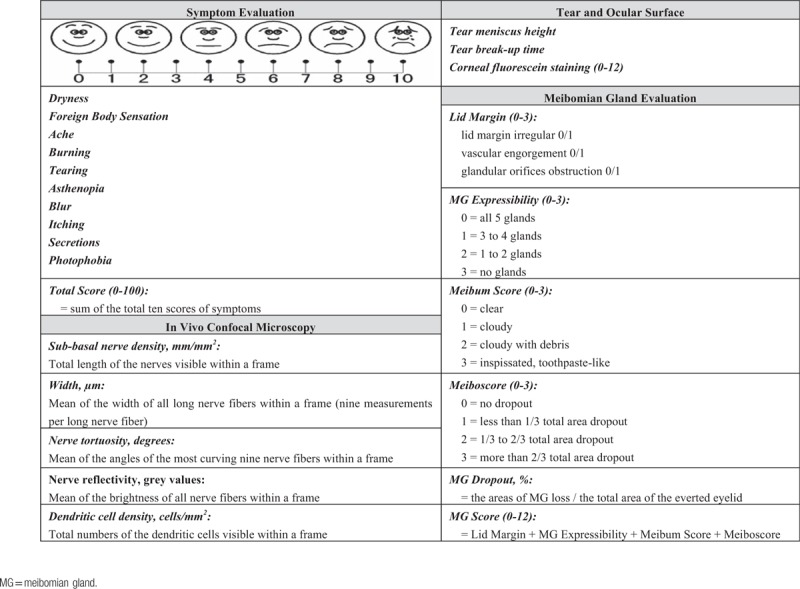
Proposed grading scales and clinical evaluation of meibomian gland dysfunction.

#### 2.2.1 MGD symptom evaluation

We used a self-assessment questionnaire based on previously reported studies^[13,16,17]^ which consists of 10 specific MGD symptoms including: dryness, foreign body sensation, ache, burning, tearing, asthenopia, blur, itching, secretions, and photophobia (Table 1). Each question was on a visual analogue scale of 0 to 10, with 0 for never and 10 for severe. The patients were asked to grade each symptom according to the degree to which it had affected their life, concerning both frequency and severity, in the last week. The grading procedure was guided by the same ophthalmologist to make sure all the patients understand the rule. Score of each divided question and total score of 10 questions (Total score) were both calculated to assess the severity of MGD.

#### 2.2.2 Tear and ocular surface evaluation

The central lower TMH and the TBUT were estimated by an experienced ophthalmologist using the Keratograph 5M (OCULUS, Wetzlar, Germany). CFS was tested by a slit-lamp (BQ900IM9900, Haag-Streit, Switzerland) and graded from 0 to 12 using a previously reported standard,^[18]^ a sum of the scores of the 4 corneal quadrants, which were scored individually as 0 (no staining), 1 (mild staining with a few scattered dots of stains), 2 (moderate staining between 1 and 3), and 3 (severe staining with confluent stains or corneal filaments).

#### 2.2.3 Meibomian gland evaluation

**Figure 1:**
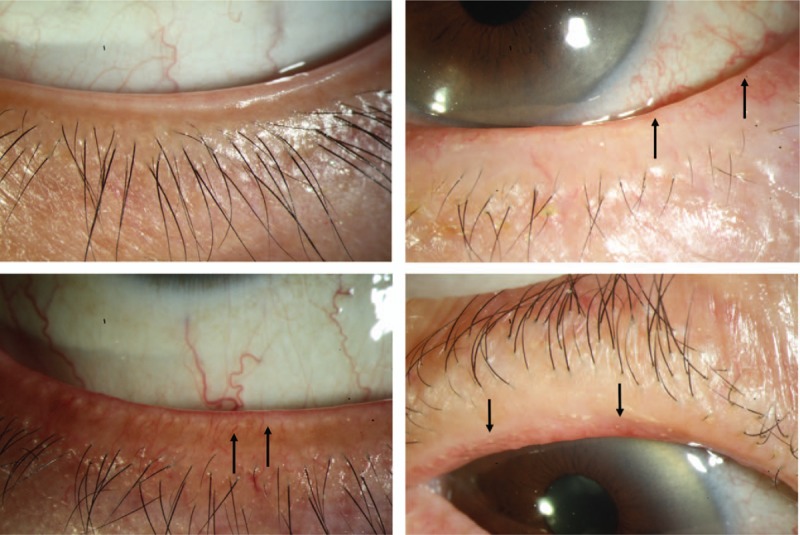
Representative images of eyelid margin. The 4 pictures represent normal (upper left), lid margin irregular (upper right), vascular engorgement (bottom left), and orifice obstruction (bottom right) of eyelid margin. Arrows indicate the typical lesion.

Slit lamp examination was performed to assess and record the severity of the 3 MG signs of the lower eyelid on a 4-point categorical scale (0–3) as reported previously.^[10,16,19]^ The abnormality of eyelid margin (Lid Margin Score) was assessed by the following 3 factors: lid margin irregularity, vascular engorgement, and glandular orifice obstruction assigned one score each. The representative images of criteria were shown in Fig. 1. Meibomian gland expression (MG expressibility) was scored according to the secretion seen in all 5 meibomian glands^[20]^: 0, all glands; 1 (3–4 glands); 2 (1–2 glands); and 3 (no glands). Meibum quality (Meibum score) was graded as follows^[21]^: 0 (clear); 1 (cloudy); 2 (cloudy with debris); 3 (inspissated, toothpaste-like).

MG morphology of the lower eyelid was observed by the Keratograph 5M (OCULUS) and further analyzed both subjectively and semi-objectively based on 1 image with optimal quality for each patient. Subjectively, the Meiboscore was defined as described by Arita et al^[22]^ using the 4-point grading scale (0–3): 0 (no dropout); 1 (<1/3 total area dropout); 2 (1/3–2/3 total area dropout); 3 (>2/3 total area dropout). Semi-objectively, MG dropout was measured as reported by Srinivasan et al^[23]^ through the same images with further processing by ImageJ 1.46r (Wayne Rasband, National Institutes of Health, Bethesda, MD; http://imagej.nih.gov/ij), an open source image analysis software, and calculated by two researchers for the average results. Briefly, it was expressed as the percentage when the areas of MG loss were divided by the total area of the everted eyelid.

Finally, we calculated the total score of the MG grading examinations mentioned above (MG score), which ranged from 0 to 12 as an integrated evaluation of the meibomian gland.

#### 2.2.4 In vivo confocal microscopy

IVCM was performed on all patients with a digital corneal confocal laser-scanning microscope (HRT II RCM Heidelberg Engineering Inc., Heidelberg, Germany, Rostock Cornea Module). Three images with optimal quality were selected for analysis. A Java-based image processing software (ImageJ), and a plug-in (NeuronJ, Biomedical Imaging Group, Lausanne, Switzerland) were used for quantification. The data including sub-basal dendritic cell density; sub-basal nerve density, width, tortuosity, and reflectivity were recorded as the mean values of 3 measurements by an experienced ophthalmologist. Details on the methods of image acquisition and parameter analysis have been reported by Antoine et al.^[24]^

#### 2.2.5 Statistical analysis

Statistical analyses were performed using SPSS version 22.0 (IBM Corp., Armonk, NY). The Kruskal–Wallis test was used to test for statistical differences among the 3 groups. The Mann–Whitney *U* test was used to determine the difference between individual pairs of groups. Correlations among variables were analyzed with Spearman coefficient. A 2-sided *P* value <.05 was considered statistically significant for all comparisons.

## 3 Results

**Figure 2:**
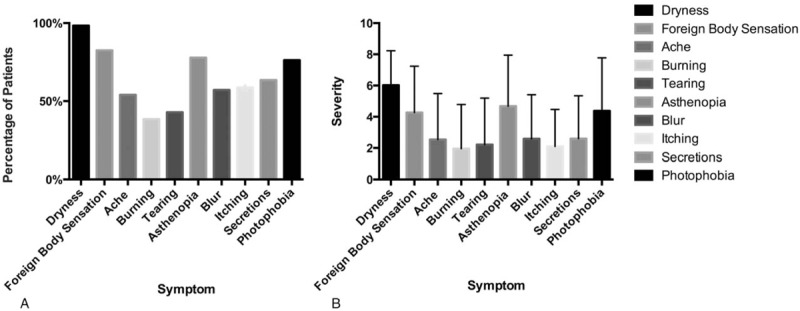
Symptoms of meibomian gland dysfunction.

A total of 63 patients with MGD (63 eyes) were enrolled with 37 patients in the mild MGD group, 19 in the moderate MGD group, and 7 in the severe MGD group. As shown in Table 2, no difference was observed among the 3 groups in terms of sex (*P* = .630) and age (*P* = .514). The symptom scores showed statistically significant differences among the groups (*P* < .001), while there was no statistical difference in the tear and ocular surface sign tests including TBUT, TMH, and CFS. Figure 2 describes the details of different symptoms in patients. Concerning prevalence, dryness (98.41%), foreign body sensation (82.54%), asthenopia (77.78%), and photophobia (76.19%) were the 4 most common symptoms reported by our patients with other symptoms <65% of the time. As for the severity, dryness (6.02 ± 2.21), asthenopia (4.68 ± 3.27), photophobia (4.38 ± 3.39), and foreign body sensation (4.27 ± 2.97) also ranked highest among the 10 symptoms with the average scores of the others being lower than 2.60.

**Table 2:**
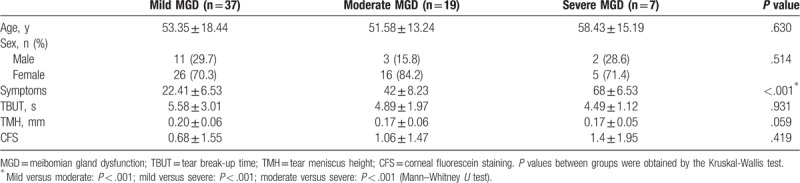
Demographics and evaluation of tear and ocular surface.

The comparison of meibomian gland assessments among groups is shown in Table 3. Patients in the severe MGD group had significantly higher scores in MG expressibility, meibum, and meiboscore than had the patients in the other groups (*P* < .05), which implied a worse meibomian gland condition. There was no obvious difference in the lid margin score among the 3 groups. However, the total MG score, including all MG tests, was still significantly different (*P* < .01), which highlights the effectiveness of the evaluation.

**Table 3:**
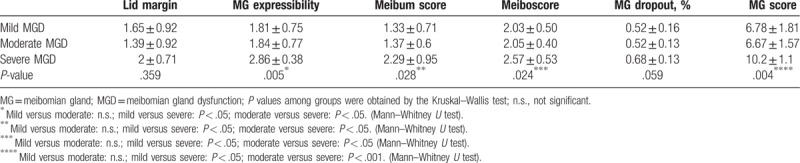
Scores of the meibomian gland evaluation.

Furthermore, IVCM was applied to assess the change in the nerves and inflammatory cells of the cornea, which may explain the symptoms of MGD. As shown in Table 4, there was significantly higher sub-basal nerve density in the mild MGD group compared with the moderate MGD group (*P* < .01). Patients in the moderate MGD group also showed higher sub-basal nerve density than did those in the severe MGD group, although the difference was not statistically significant. Regarding nerve reflectivity, patients with severe MGD had the highest grey values (*P* = .005) while no difference was observed between the other 2 groups. There was no significant difference in nerve width, nerve tortuosity, and dendritic cell density among the groups.

**Table 4:**
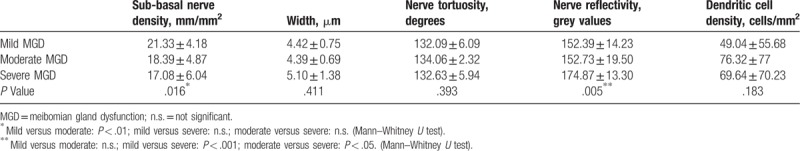
IVCM analysis of sub-basal nerve and dendritic cell parameters.

Finally, we calculated Spearman correlations to explore the relationship between the symptoms and signs of tear, ocular surface, and meibomian glands. As shown in Table 5, TMH was negatively correlated with foreign body sensation (*r* = –0.258, *P* = .04), burning (*r* = –0.274, *P* = .03), and the total symptom scores (*r* = –0.352, *P* = .005). Significant correlations were also observed between foreign body sensation and MG Expressibility (*r* = –0.274, *P* = .03) and between tearing and Meibum (*r* = 0.349, *P* = .005). Blur and itching were both negatively correlated with TBUT (*r* *=* –0.258, *P* = .04 and *r* = –0.258, *P* = .04, respectively). Blur was positively correlated with MG expressibility and MG score (*r* = 0.390, *P* = .002 and *r* = 0.278, *P* = .03, respectively). And the secretions were positively correlated with the Meiboscore and MG dropout (*r* = 0.303, *P* = .02 and *r* = 0.295, *P* = .02, respectively).

**Table 5:**
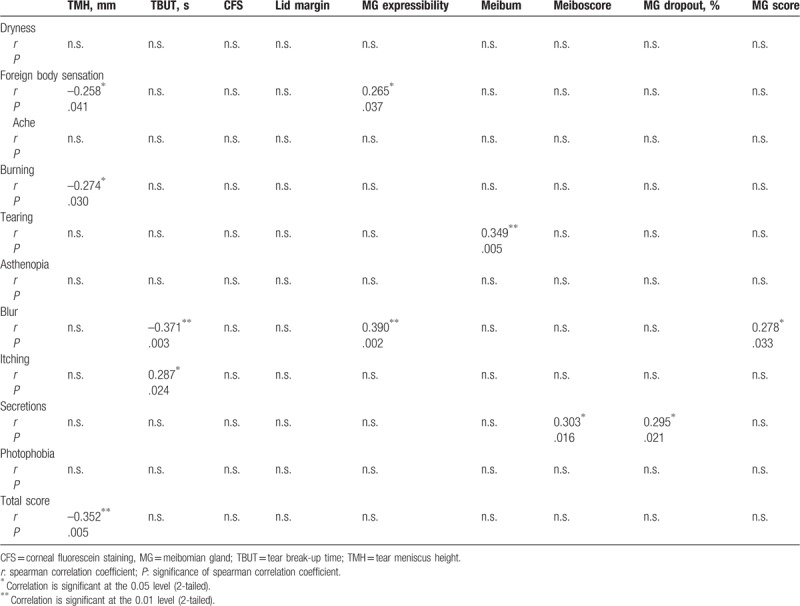
Correlations between symptoms and ocular signs.

Moreover, correlation analysis was also used for the IVCM results. Table 6 describes the relationship between the IVCM parameters and the symptoms while Table 7 describes this relationship for the signs. The total score of the symptoms was correlated with sub-basal nerve density (*r* *=* –0.374, *P* = .003), nerve reflectivity (*r* = 0.332, *P* = .008), and dendritic cell density (*r* = 0.288, *P* = .022); while the MG total score was correlated with nerve reflectivity only (*r* = .265, *P* = .043). Specifically, sub-basal nerve density showed significant correlations with foreign body sensation (*r* = *-*0.308, *P* = .014), blur (*r* *=* *–*0.263, *P* = .037), secretions (*r* *=* –0.255, *P* = .044) and photophobia (*r* = 0.263, *P* = .037); while nerve reflectivity showed significant correlations with dryness (*r* = 0.348, *P* = .005), foreign body sensation (*r* = 0.373, *P* = .003), burning (*r* = 0.265, *P* = .036), secretions (*r* = 0.303, *P* = .016), and photophobia (*r* = 0.300, *P* = .017). Dendritic cell density was positively correlated with both burning (*r* = 0.271, *P* = .032) and secretions (*r* = 0.358, *P* = .004). Conversely, significant correlations were also observed between sub-basal nerve density and TMH (*r* = 0.393, *P* = .001) and between nerve density and MG dropout (*r* = 0.320, *P* = .012).

**Table 6:**
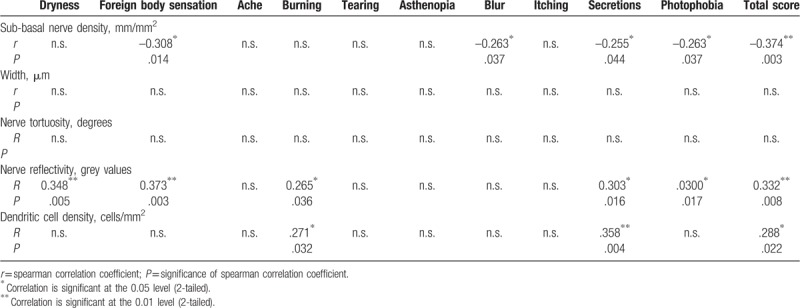
Correlations between IVCM results and symptoms.

**Table 7:**
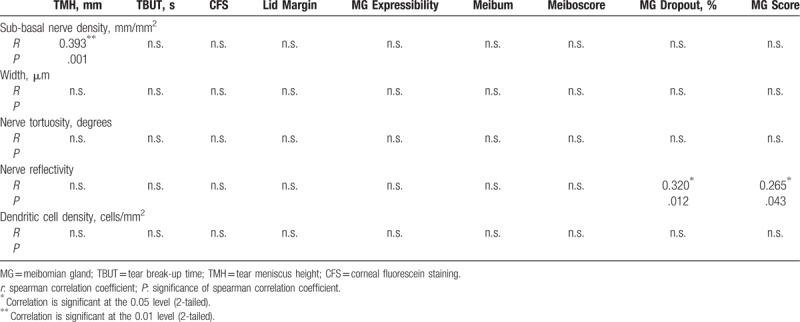
Correlations between IVCM results and ocular signs.

## 4 Discussion

In this study, we proposed a comprehensive grading scale to evaluate the clinical manifestations in patients with MGD with varying severity from an integrated perspective concerning impairment of the whole ocular surface morpho-functional unit. Further, we explored the correlations between specific symptoms, signs, and the IVCM parameters. We found dryness, foreign body sensation, asthenopia, and photophobia as the most common and severe symptoms affecting the daily quality of life of our MGD patients. Further, our grading scale proved helpful to differentiate alterations in MG signs between severe MGD group and others while in IVCM results between mild MGD group and others. Moreover, the study found significant correlations between the IVCM analysis results and signs and symptoms of MGD, emphasizing the necessity of evaluating MGD from an integrated perspective. These results and grading system may help us better assess the disease in a precise manner and further offer targeted treatment to patients of varying severity, accordingly.

The International Workshop on MGD indicated the urgent need for symptom assessment in a more refined way, but this has been the focus of few studies.^[25]^ We used a grading system on a visual analogue scale, similar to the one used for pain assessment, including 10 specific symptoms based on previously reported studies.^[13,16,17]^ The results show that dryness, foreign body sensation, asthenopia, and photophobia are the most common and severe complaints in our patients. Thus, we suggest that ophthalmologists should consider to evaluate MG signs and cornea conditions when encountering patients with those symptoms. Given the previous study which reported that symptoms alone for diagnosis provided an area under the receiver operating characteristic curve of 0.95,^[16]^ it is necessary to assess the MGD symptoms in detail to differentiate the disease and further benefit from precise treatment targeted toward specific symptoms on an individual basis.

The validity of our grading system was proven through the significant differences found in MG signs and the IVCM parameters among different groups. We observed worse MG condition in all signs except Lid Margin, including MG expressibility, meibum score, meiboscore, MG dropout, and MG score, which varied consistently with the severity level of the disease. As for tear and ocular surface signs including TMH, TBUT, CSF, there were no significant differences among the 3 groups which were in accordance with the findings of previous studies.^[14,26–28]^ However, our results indeed show the trend that the impairment is consistent with the severity of MGD. As the severity increases, the TBUT and TMH is shorter while the CFS is higher. We suppose further studies including more patients may find significant results. In our study, concerning the MG signs, only the difference between the severe group and others reached statistical significance while the mild and moderate group did not. As for the IVCM results, we found sub-basal nerve density decreased with more severe disease levels and nerve reflectivity in the severe MGD group showed the highest grey values among the 3 groups. Changes in IVCM parameters regarding corneal nerves and dendritic cells is a subject of debate.^[11,29,30]^ Although the mainstream opinion supports that there is decrease in sub-basal nerve density and nerve reflectivity while increase in nerve width and tortuosity and dendritic cell density in MGD,^[24,31,32]^ some studies have found opposite or insignificant change in the related parameters.^[33,34]^ However, those results were derived from the comparison between patients with MGD and a healthy control group which holds a little difference from our study focusing mainly on various levels of severity in MGD. Briefly, we attribute all the similarities and differences above to the progression of the disease.^[8,14,19,26]^ We assume that the IVCM changes in MGD occur earlier than the MG signs change.^[35]^ Thus, the most obvious altered MG signs were in the severe group and not in the others while the most obvious change in sub-basal nerve density was in the mild group representing an early impairment. Moreover, since there is decompensation followed by compensation during the procession of MGD pathophysiological pathology, the fluctuations in the other IVCM parameters along with the severity could be well explained.

Remarkably, researchers have recently proposed a new concept of one unit, that is, the “meibomian gland and ocular surface unit,”^[6,36]^ “the ocular surface morpho-functional unit,”^[37]^ or the “lacrimal functional unit”^[24,38]^ which comprises mainly the meibomian glands, the tear film, ocular surface, and other related organs or materials. Such definition empowers us to understand MGD at a new level and further offers us a more thorough platform to study the disease. Given the current research controversy, the birth of the new notion is inevitable for the following reasons. First, there are no reliable evaluation tests or systems for MGD recognized as the gold standard both for diagnosis and grading and the existing signs of MGD often do not correlate with symptoms of discomfort, not to mention other tests using the equipment.^[8]^ Second, it is the pathophysiological progression of the disease that calls for a more integrated system for its diagnosis and treatment.^[19]^ MGD is characterized by abnormality of terminal duct obstruction and/or qualitative or quantitative changes in glandular lipid secretion, which will further affect the tear quality/quantity and eventually impair ocular surface homeostasis.^[8]^ At the same time, the injured ocular surface which has triggered the inflammatory cascade by MGD will in turn damage the meibomian gland.^[6,36]^ Once this vicious circle has formed, it would be unilateral to evaluate the disease only from limited aspects rather than the entire complex system. According to this perspective, we further explored the correlation of the related parameters from an integrated perspective including: MGD-specific symptoms, meibomian gland signs, tear and ocular surface signs, and IVCM analysis of the sub-basal corneal nerves and dendritic cells. The reason we contained the IVCM for corneal nerves and dendritic cells was considering the hot spot of the pathophysiology of the disease to be related to immunology and neurology.^[5]^ Despite the limitation that we could not inspect the meibomian innervation and the inflammatory cytokines directly, some studies on both animals and humans have proven the homology and the similar secretion of peptides between the meibomian and the cornea nerves, which could explain the symptomatology of MGD to a great extent.^[39]^ In general, we found that the total symptom score was negatively correlated with sub-basal nerve density and TMH, while positively correlated with nerve reflectivity and dendritic cell density. The MG score was positively correlated with nerve reflectivity, and TMH with sub-basal nerve density. These results proved the consistency of our grading system and the correlations of IVCM with the total scores of both symptoms and signs also representing the validity of evaluating the severity of MGD. Despite the non-matched data between the signs and symptoms as previously reported,^[7]^ the IVCM results showed good correlation with the related parameters, which emphasized the necessity of the tests. In detail, the decreasing sub-basal nerve density as well as the increasing nerve reflectivity and dendritic cell density varied along with the severe level of the disease that could explain the potential immune and neurological mechanisms of MGD symptoms such as foreign body sensation, burning, secretions, and photophobia as reported previously.^[40,41]^. The insignificant results of nerve width and tortuosity, which contradicted the results of previous studies^[24,32]^ might be attributed to the disease progress as mentioned above, since the patients in our study had varying severity and they may have been in either the compensation stage or the decompensation stage of the disease. As for the signs, TMH had a good correlation with many symptoms and IVCM, while CFS did not, which implies that the tear, and not the ocular surface, was injured in our patients. This disparity might also be due to different populations in the study, enrolling less MGD patients at the severe stage. To our knowledge, we are the first to use the corneal IVCM results of nerves and inflammatory cells to explain the symptoms in MGD of varying severity, and to date no study has evaluated MGD severity using an integrated grading scale concerning the whole manifestation of the ocular surface.

Inevitably, this study has some limitations: First, we included few patients with severe MGD which may cause bias when comparing the differences among the 3 groups. Second, the inclusion criteria based on China's guideline for MGD patients were quite loose to differentiate MGD dry eye from other cause of dry eye. Future studies should enroll more patients with varying severity using more specific criteria. Third, the symptom grading scale in our study is of personal heterogeneity. Thus, it should be better validated and evaluated in further longitudinal investigation, which is now in progress.

In conclusion, we have developed a comprehensive grading scale in line with the tenets of precise medicine which was found to be suitable for evaluating the clinical manifestations in MGD with varying severity. The relationship between the specific symptoms, signs, and corneal IVCM results of nerves and inflammatory cells concerning whole ocular surface impairment could help elucidate the disease pathophysiology and further be used for thorough evaluation or targeted treatment of MGD in the future.

## Author contributions

**Data curation:** Jiayu Fu.

**Formal analysis:** Jiayu Fu, Yilin Chou.

**Funding acquisition:** Xuemin Li.

**Investigation:** Xuemin Li.

**Methodology:** Yilin Chou, Ran Hao.

**Project administration:** Xuemin Li.

**Writing – original draft:** Jiayu Fu, Yilin Chou, Ran Hao.

**Writing – review & editing:** Jiayu Fu, Yilin Chou, Ran Hao, Xiaodan Jiang, Yushi Liu, Xuemin Li.

Jiayu Fu: 0000-0002-6838-582X.

Yilin Chou: 0000-0003-0945-2807.

Hao Ran: 0000-0001-9632-4138.

Xiaodan Jiang: 0000-0003-2448-4815.

Yushi Liu: 0000-0001-5090-3927.

Xuemin Li: 0000-0002-4018-9974.

Yushi Liu orcid: 0000-0001-5090-3927.
